# From Drainage to Strategy: A Call for Standardization in Necrotizing Pancreatitis

**DOI:** 10.1002/ueg2.70144

**Published:** 2025-11-18

**Authors:** Giuseppe Dell’Anna, Antonio Facciorusso

**Affiliations:** ^1^ Gastroenterology and Gastrointestinal Endoscopy Division IRCCS San Raffaele Hospital Milan Italy; ^2^ Gastroenterology and Gastrointestinal Endoscopy Division IRCCS Policlinico San Donato San Donato Milanese Italy; ^3^ Faculty of Medicine, Department of Experimental Medicine Gastroenterology Unit University of Salento Lecce Italy

**Keywords:** acute necrotizing pancreatitis, acute pancreatitis, percutaneous drainage, walled‐off pancreatic necrosis

Acute necrotizing pancreatitis (ANP) remains a clinically challenging condition in gastroenterology [1]. With the expanding use of lumen‐apposing metal stents (LAMS) and dedicated devices, endoscopic ultrasound (EUS)‐guided interventions increasingly offer minimally invasive solutions even for deep or complex necroses, reducing the need for percutaneous routes [2]. Although advances in minimally invasive interventions have transformed its management, the optimal use of percutaneous drainage—alone or in combination with endoscopic techniques—remains an unsettled issue [3]. Vornhülz and colleagues from the DRACULA study group present the largest multicentre retrospective analysis to date of current practices in percutaneous drainage for necrotizing pancreatitis, revealing striking variability across 29 tertiary centers and offering valuable insights into real‐world management patterns [4].

The authors examined 585 patients with necrotizing pancreatitis who underwent at least one percutaneous drainage procedure between 2016 and 2022. Their analysis focused on flushing regimens, catheter caliber, and the combination of percutaneous and internal drainage—so‐called dual‐modality drainage (DMD). Surprisingly, flushing, whether performed with saline or antibiotic solutions, was not associated with improved outcomes in terms of hospital stay or mortality. Conversely, patients receiving DMD experienced significantly lower mortality compared with those managed by percutaneous drainage alone [4].

This finding supports an evolving concept: percutaneous drainage should not be regarded as an isolated or “competing” modality but rather as part of a broader, integrated strategy. Over the past decade, evidence has consistently favored a minimally invasive step‐up approach, prioritizing internal EUS‐based drainage whenever feasible [5]. When anatomical constraints preclude an endoscopic route, percutaneous drainage provides a valuable adjunct to reduce sepsis, control collections, and serve as a bridge to internal access. The DRACULA data reinforce this complementary role, highlighting that outcomes improve when both modalities are combined—a message that resonates strongly with the current trend toward hybrid, multidisciplinary management of pancreatic necrosis [4].

Another key message emerging from this study is the need for standardization. The authors observed remarkable heterogeneity in almost every aspect of percutaneous management: flushing frequency varied from once daily to continuous irrigation, daily volumes ranged from 30 to 750 mL, and solutions included saline, antiseptics, or antibiotics. Yet, none of these variations correlated with improved outcomes. This inconsistency underscores the absence of evidence‐based recommendations in existing guidelines—an unmet need the DRACULA study compellingly exposes [6].

The clinical implications are clear. First, indiscriminate or empiric flushing, especially with antibiotic or antiseptic solutions, lacks evidence and may even contribute to antimicrobial resistance or higher costs. Simple saline irrigation, when performed, appears sufficient and safer. Second, catheter size should be tailored to anatomy and drainage effectiveness rather than driven by dogma: large‐bore catheters, while facilitating lavage, may increase the risk of fistula formation and delayed recovery [3]. Third, the observed survival advantage with DMD highlights the importance of multidisciplinary coordination between interventional radiologists, endoscopists, and surgeons—an essential component of modern pancreatic care that is still inconsistently implemented across Europe.

From a methodological standpoint, DRACULA is also noteworthy. By adopting restricted mean survival time (RMST) analysis and propensity‐weighted adjustments, the investigators provide robust statistical evidence in a challenging retrospective setting. Nevertheless, as the authors acknowledge, causality cannot be inferred, and residual confounding is likely, particularly since patients requiring flushing may represent more severe disease phenotypes.

Looking ahead, several avenues for research emerge. Recent evidence on EUS‐guided drainage has proposed a standardized classification system (Quadrant–Necrosis–Infection, QNI), incorporating the extent of involvement, degree of necrosis, and presence of infection to stratify collections into low‐ and high‐risk categories and to identify predictors for escalation to a step‐up approach [1, 7, 8, 9]. This kind of approach should not be limited to EUS‐guided management, but extended to a comprehensive standardized view of necrotizing pancreatitis treatment. Future studies should also identify predictors for patients who may benefit from combined or multi‐gateway drainage approaches, integrating morphological, microbiological, and clinical parameters [10]. However, until such techniques are universally available, standardizing percutaneous management remains essential to ensure equity of care and reproducibility of outcomes.

Ultimately, the DRACULA study is a reminder that in necrotizing pancreatitis, how we drain may be less important than how thoughtfully we integrate available options. Standardization of percutaneous protocols and closer alignment with endoscopic approaches could represent the next step forward—turning drainage from an art of improvisation into a reproducible, evidence‐based component of care. The study's message is both simple and powerful: the future of pancreatic necrosis management lies not in debating techniques, but in orchestrating them.

## Funding

The authors have nothing to report.

## Conflicts of Interest

The authors declare no conflicts of interest.

## Data Availability

No new data were generated or analysed in support of this editorial.
